# Task shifting or shifting care practices? The impact of task shifting on patients’ experiences and health care arrangements in Swaziland

**DOI:** 10.1186/s12913-016-1960-y

**Published:** 2017-01-10

**Authors:** Thandeka Dlamini-Simelane, Eileen Moyer

**Affiliations:** 1 Department of Anthropology, University of Amsterdam, Nieuwe Achtergracht 166, 1018 WV Amsterdam, Netherlands; 2 1493 Lukhasi Street, Ext. 11 Thembelihle, P.O. 6231, Mbabane, H100 Swaziland

**Keywords:** Task-shifting, Expert clients, Patient friendly services, Global health, Implementation science, Swaziland

## Abstract

**Background:**

In the quest to achieve early HIV treatment goals, national HIV treatment programmes dependent on international funding have been dramatically redesigned over the last 5 years. Bottlenecks in treatment provision are conceived of as health system problems to be addressed via structural and logistical fixes (routine HIV testing, point-of-care equipment, nurse-led antiretroviral treatment initiation, and patient tracking). Patient perspectives are rarely taken into account when such fixes are being considered. Patients’ therapeutic experiences often remain at the periphery during the planning stage and are only considered within the context of monitoring and evaluation audits once programmes are up and running.

**Methods:**

Ethnographic research was conducted in five clinics in Swaziland between 2012 and 2014. Participatory approaches were used to collect data; the first author trained as an HIV counsellor in order to collect observational data on the continuum of care, and conducted in-depth interviews with interlocutors involved at the different phases.

**Results:**

Although recently adopted global HIV strategies have proven effective in scaling up treatment in Swaziland, our research demonstrates that the effort to expand services rapidly and to meet donor targets has also undermined patients’ therapeutic experiences and overtaxed health workers, both of which are counterproductive to the ultimate goal of treatment scale-up. This article provides a perspective beyond the structural elements that impede universal treatment, and explores patient views and experiences of the strategies adopted to support further treatment expansion, with a particular focus on the shifting of key care and logistical tasks to expert clients.

**Conclusion:**

We argue that in the quest to achieve universal early access to treatment, both donors and states must go beyond strengthening health systems and strive to enhance the quality of patient experiences and take seriously health worker limitations.

## Background

Research for this article was collected within the framework of a larger research and intervention project in Swaziland known as MaxART (Maximising ART for Better Health and Zero New HIV Infections).^1^ The project was designed to encourage researchers, activist organizations, and implementation scientists to work collaboratively with the Swazi Ministry of Health to: 1) identify and minimise structural factors to treatment access, 2) expand HIV testing and counselling (HTC), 3) investigate the everyday realities of people living with HIV (PLHIV) seeking treatment, and 4) ensure that the ambitious expansion of HIV care and treatment was carried out within a human rights framework [1]. The ultimate aim of the project was to assist in the massive expansion of antiretroviral treatment (ART) in Swaziland in order to test the evidence from the HPTN 052 trial, which hypothesised that widespread use of ART would prevent new HIV infections at a population level [2]. To achieve this ambitious aim, various globally instigated processes, principles, and strategies were introduced and intensified in Swaziland, including the shifting of several tasks, such as provider-initiated HIV testing and counselling, nurse-led ART initiation, and the involvement of PLHIV in the delivery of HIV care [1].

Beyond the structural factors limiting the expansion of ART that the MaxART project aimed to eliminate, our research identified several other important factors that limited individual uptake of services. Even when health facilities were easily reachable in terms of distance, and fully staffed and equipped, some people still did not utilise the services as expected. This article describes such other factors that impede treatment, and explores patient experiences of and perspectives on the processes and strategies adopted to support the further expansion of treatment, with a particular focus on the shifting of key care and logistical tasks to expert clients.^2^ Introducing lay health workers without biomedical training into clinical spaces brought about changes in care practices and challenged institutional hierarchies in the consultation and counselling rooms, in the laboratory, and among differently positioned health workers and patients. Our research examined these changes that occurred as expert client task shifting was implemented, and how the changes shaped patients’ therapeutic experiences. In this article, we highlight the diversity of new care practices that have been incorporated at the ground level of ART delivery and discuss the actors most affected by task shifting.

Most monitoring and evaluation frameworks track results: number of people tested, enrolled, and retained in treatment programs. By focusing on everyday practices of care within the context of ART clinics we provide important information about the ways that gaps in the HIV care continuum are created in situ. Our approach, combining ethnographic observation and qualitative interviewing, allowed us to examine both the social and logistical processes involved in achieving these numbers, and to draw attention to the intensive labour of the non-medically trained workers who assist patients to navigate these processes. This article builds on a growing body of qualitative research on task shifting.^3^ It has shown to be a cost-effective response to HIV in Zambia [3] to have enhanced the quality of HIV care in Malawi and Mozambique [4], and to have enhanced the experience of health workers [5]. In Ethiopia an expansion in uptake of counselling and ART was attributed to task shifting [6]. With a focus on the new processes and procedures introduced by task shifting, we examine changes in the constellation of HIV care and how these have shaped the therapeutic experiences of patients and the quality of care.

### History of task shifting

In 2006, during the High-Level Meeting on HIV/AIDS for the United Nations General Assembly, the UN committed to work towards universal access to ART by 2010. At the time, there were glaring health system weaknesses in many of the countries hardest hit by HIV, particularly with regard to human resources. The ‘treat, train, and retain’ plan was devised by WHO [7] as a response. The aims of the plan were quadruple: prevent new infection among health workers, treat infected workers, expand capacity of existing health personnel, and add new cadres of workers [8]. Task shifting was specifically identified as a strategy to resolve the last two issues. The shifting of health care-related tasks to lower cadres of health workers was formally advocated by UNAIDS in the context of HIV treatment scale-up in 2007 [9].

At the time, task shifting in the context of HIV-related health services was already being practiced in Swaziland. Lay health workers, predominantly PLHIV, were taking over some nursing tasks, including HIV counselling, whilst nurses took over some of doctors’ duties to start patients on ART. Beyond the context of HIV, nurses had long performed tasks that were officially reserved for medical doctors, for example administering intravenous medicines and doing sutures.

In 2006 the Swazi Ministry of Health decentralised ART provision to the lowest levels of the health system, which resulted in the need to expand the health workforce. The health system was already facing a formidable human resource crisis due to a combination of factors including ‘brain drain’, with Swazi medical professionals often migrating, and a low health worker output in the country. The total number of nurses graduating annually in Swaziland was only 80, far below the numbers required for treatment expansion. Two years after ART became available for free in public health facilities in Swaziland, Kober and Van Damme [10] examined health-related human resources and found the situation dire: 44% of physician posts, 19% of nurses’ posts, and 17% of nursing assistant posts were vacant. This shortage was all the more poignant in the context of increasing demand for HIV-related testing, counselling, and treatment services that came with the nation’s efforts to promote universal treatment access.

In order to meet these demands, a new cadre of lay health workers, expert clients, was introduced in health facilities. A year after the use of expert clients in task shifting was officially instituted in the country’s ART programmes, Samb and colleagues [8] reported a more promising health workforce landscape with ratios nearing the global recommendations for expanding ART services: 1 doctor to 1,135 patients, and 1 nurse to 64 patients (18 doctors and 320 nurses for the 20,427 HIV-positive persons in care at the time). Although addressing the human resource shortage was not the only change made to the HIV-related medical system, the improved human resource situation significantly benefited the ART programme, which recorded tremendous success within a few years. By 2010, 4 years after the introduction of task shifting and expert client services, 64% of those eligible for treatment were receiving it (Ministry of Health Strategic Information Department. Monitoring the STI, HIV and TB Response Report. unpublished); by the end of 2012, 91% of eligible HIV-positive people were on ART (NERCHA 2014). In 2014, Swaziland was applauded by UNAIDS for making rapid progress toward achieving universal access to ART, and task shifting of key care-related tasks to lay health workers was named as a major factor in achieving that success [11]. Despite this well-deserved praise, some barriers continued to impede treatment uptake among some people, including distance to health facilities, limited infrastructure, and a limited capacity in some ART clinics [9]. While the high enrolment numbers were laudable, we argue that the success concealed the experiences and challenges many patients and health workers faced in the quest to expand treatment access quickly.

### Who is an expert client?

In Swaziland, an expert client is someone who is living with HIV and actively taking antiretroviral medications, who has demonstrated good adherence to treatment, encourages others living with HIV to seek treatment, and is willing to disclose his or her status and share the experience of living with HIV with other patients. Seen as model patients, expert clients live a ‘normal life’ despite HIV infection. They are hired as full-time, minimally paid volunteers to work within health facilities as health workers. In the clinics where we conducted research, expert clients had initially been engaged to provide counselling. However, over the years as the need for more health workers grew in the context of treatment expansion, the tasks of expert clients expanded to include: HIV testing, pre- and post-test counselling, enrolment of patients into care, booking patients for appointments, adherence counselling for patients on ART, and patient tracking. There are two ways to become an expert client in Swaziland: one can respond to an advertisement, which is normally placed on a clinic’s bulletin board and lists the above mentioned attributes, or an ART nurse can identify an adherent patient as a good candidate. Additionally, each candidate undergoes an interview and an offer is made to the best candidate. Before expert clients start working in health facilities, they are trained for 6.5 days following a curriculum which consists of 14-units: introduction to HIV, HIV basics, paediatrics and HIV, impact of HIV, role of expert clients, communication and counselling skills, HIV care and treatment, adherence to treatment, stigma and disclosure, positive living, record keeping, referrals, facility-community linkages and a practicum.

## Methods

This article draws on 25 months of ethnographic research (June 2012–July 2014) conducted in Swaziland to examine the HIV continuum of care in public health facilities. The aim was to identify the social and logistical dynamics that contributed to delayed uptake of ART and for patients dropping out of HIV-related care. To better understand the delays and disruptions occurring in the care continuum, we focused our investigation on the health system itself, paying attention to logistical and human resource issues. As part of the main study, we hypothesized that care and treatment access were shaped by factors beyond the clinic, however, we also examined wider sociocultural norms that shaped access to care and treatment-for example norms relating to gender, marital status, and age-as well as the perspectives of individual patients [12].

Data was collected from five clinics. The first author was attached to three clinics (referred to as *fundza* sites) as an intern in an HIV counselling course (HTC); two additional clinics (*gusta* sites) were also selected for in-depth ethnographic research. Two of the *fundza* research sites were located in the city centre of the, and one was located in a peri-urban area. The two sites located in the city were highly-resourced hospitals that were fully equipped and served as ART initiation sites. Because they received private funding, they were not entirely depended on donor funding or government. The *gusta* sites were not in urban setting; one was located at the periphery of the capital city and the other in a rural area approximately 200 km from the capital city. They were both community clinics and primarily dependant on government funding and donor supported. Findings presented in this paper were derived from participant observations and time series in-depth-interviews (IDIs) with patients who were eligible but did not initiate ART in 2011. At the onset of fieldwork, the first author enrolled in a 5-week HTC course and trained as an HIV counsellor. Two weeks of classroom training on the theoretical aspects of HIV counselling were followed by 3 weeks of practicum providing HTC services to patients testing for HIV. At completion of the training the first author became a certified HIV counsellor, which permitted her to be part of the counselling team in three clinics where she collected observational data.

### Participant observation

Attending the counselling course allowed the first author to gain first-hand experience as a health worker providing HIV services, to compare counselling services in practice with counselling as taught in the classroom, and to gain insight into the factors that led counsellors to circumvent guidelines. Her positioning within the health system also provided a unique practitioner perspective on issues that contributed to patients dropping out of care. Working in the counselling department allowed for the observation of patients at multiple points in the care continuum because counselling was provided in the context of testing, in the lead-up to treatment initiation, and in follow-up visits tracking adherence. During the practicum period, the first author provided various types of counselling to 80patients, including pre- and post-test HIV counselling and adherence counselling.

At the two additional clinics (*gusta*), the first author observed 136 HIV counselling sessions over a period of 20 months. During these observations, she took detailed notes to record the type of information provided to patients following HIV diagnosis, the manner in which it was delivered, the behaviour of patients in the counselling room, and how counsellors interacted with returning patients who had missed clinic appointments. Additionally, the first author assisted counsellors as they executed their daily duties, helping to track patients, sorting patient files, filling registers, and booking patients for future appointments. Research in these two clinics allowed the first author to compare working as a counsellor with the practices of health workers in routine clinical settings.

Observations went beyond the counselling room to include the entire ART department in each of the two (*gusta*) clinics where in-depth research was conducted. The first author was on-site full time at each clinic for 2 months, observing clinical operations in all departments, including the laboratory, the ART nurses’ station, consultation rooms, and the dispensary where she assisted in the packing of medicines. After the 2 months, she continued to return periodically to each clinic to follow up with particular patients and health workers until the end of the research period.

The first author also completed direct observations of clinic logistics: the management of queues, tracking and management of clinic defaulters, and the keeping of client registers. These, together with client health cards, were studied to collect an overview of patient histories and demographics. In the context of participant observation, numerous informal conversations were held with ART staff, including nurses, expert clients and the phlebotomist. In addition, many other conversations were overheard in both formal and informal contexts (i.e., lunch or tea breaks, rides home) among lay workers, nurses, other clinic staff, and patients.

### In-depth interviews

In-depth semi-structured interviews were conducted with participants purposively selected from two sources: patients observed in the counselling room and patients identified as ‘defaulters’ by counsellors. HIV-positive persons eligible to begin ART were scheduled for a series of counselling appointments. If a client failed to show up for an appointment, the counselling staff put the client’s file into a stack of defaulters. From those files, we narrowed the pile to include only those who had been scheduled to start ART in 2011 to tally with the beginning of the MaxART project. Counsellors routinely telephoned defaulters to ask the reason for the missed appointment and then reschedule. When patients reported back to the clinic, they were approached and invited to participate in the study after being informed of the study parameters and giving consent by signing a consent form.

Selection criteria limited participants to patients who had failed to return to the clinic for scheduled appointments, and patients who verbally stated not wanting to take ART medications as their reason for defaulting. Of the 93 patients who met the selection criteria, 71 agreed to be interviewed and a total of 104 interviews were conducted. Of the 22patients who were not interviewed, several refused and others proved impossible to track.

Interviews were conducted at a place and time convenient to the participants, and were conducted in siSwati, the first language of both the first author and the participants. Interviews began with informal conversations. Patients were then invited to speak about their overall experiences at the clinic in general and specifically why they had not returned for previously scheduled appointments. Data collection was an iterative process that allowed for crosschecking findings with subsequent research subjects to come up with themes until saturation was reached. Interviews lasted between 30 and 120 min, they were transcribed in siSwati, and then translated into English by the first author.

### Data analysis

All observational data from the counselling course was recorded as field notes, especially system-related dynamics and processes that would potentially cause patients to drop out of care. The data was analysed daily and, following the second week of the practicum, was categorised into themes. Recurring process flaws observed in the second week were matched with corresponding themes already identified. In the two ethnographic research sites, the analysis framework developed was matched and contrasted with recurring themes until the research process ended. Themes that emerged from the observational data were explored further via in-depth interviews with patients and health workers to seek the perspectives of both. NVivo was used for content analysis when data collection was completed to identify recurring and overarching themes. The themes were also analysed per research site to identify convergences and divergences in data.

## Results

The formal introduction of task shifting in HIV clinics in Swaziland introduced a new cadre of health workers and multiple new processes and strategies as clinic functions grew and the various tasks of doctors and nurses expanded and shifted. Observations allowed us to track the variety and extent of the changes in clinic policy and practice that accompanied the formal introduction of task shifting. This section highlights the ways that task shifting affected health care delivery and practices in the clinics where it was implemented and, further, how it affected patient experiences. Task shifting in the study sites entailed nurses initiating ART, a service previously rendered by doctors, and nurses delegating tasks to expert clients. The tasks shifted by nurses to expert clients included HIV testing, pre- and post-test counselling, enrolment of patients into care, adherence counselling for patients on ART, and patient tracking. Previously, expert clients working in clinics as PLHIV volunteers had provided psychosocial support to newly diagnosed HIV-positive people; with task shifting, they had to double as HIV counsellors and nursing assistants.

### Too many hands, too many processes

Contrary to expectations, our data suggests that task shifting has not resulted in improved quality or more patient-friendly services. Patients must now pass through ‘too many hands and processes’ before they finally get the services they seek. I met Sibakhe, a 27-year-old man, 2 weeks after he was tested and diagnosed with HIV, who was frustrated that he still was not on ART. He had wanted to start ART immediately after diagnosis, but was told he needed to first go through a lengthy process to prepare him for ART. Expressing his dissatisfaction with the process, he told me:

Can you imagine, you have a headache and you feel the pain now, but instead of being given medication now you are told to do 1, 2, and 3 first. They told me I am HIV positive, the cause for my running stomach, so I need to take treatment. I said I was ready to take treatment home that very day but they said that it doesn’t work like that (personal communication, September 2012).

Similarly, Tukisa had learned of her HIV status 6 months before we met. She had been waiting for 2 months for the clinic to call her with her chemistry results from the national laboratory. This resulted in a bottleneck in her treatment trajectory as the test results were a prerequisite for ART initiation.

When I discovered I was HIV positive it was a difficult time in my life. It took me 5 months to finally agree to start treatment. I became aware of people I know who are on ART. That is when I counselled myself and I went straight to the clinic. The process was explained to me, that I had to do various tests and three counselling sessions before I could start treatment. I finished the counselling, and the results from the national lab were pending. I became distraught when 1 month passed and there was no word from the clinic about my results. I went back to the clinic three different times to check my results and they were still not back. It has been more than 2 months waiting for them and I am worried whether I will ever initiate ART and get my health back like others taking ART. (personal communication, November 2012)

These interview excerpts show the frustration that patients often reported. From the patients’ perspective, they were ready to start treatment so they could begin healing, but the mandated process for patients newly diagnosed with HIV involved numerous steps that are spread out over several clinic visits. From the patient’s perspective, postponing treatment to a future date does not make sense, especially when they are not feeling well and are ready to start taking medicines. The delays in HIV treatment initiation are tied to procedure that stipulate three counselling sessions prior to starting treatment, as well as delays in getting laboratory results. Such bottlenecks are unique to HIV treatment in Swaziland, and have been exacerbated by the country’s test and treat initiatives. The solutions-introducing a new cadre of workers and mandating a multi-step treatment initiation process, with different health care workers responsible for each step-have led to a situation where patients have to pass through too many hands to access treatment. Although these solutions were put in place to resolve the high volume of patients, unfortunately they also frustrate patients, resulting in delayed treatment uptake for those ready to start treatment as soon as possible. In some cases, frustrations with the health system, combined with the social and economic challenges of multiple clinic visits, lead to patients dropping out of care altogether or choosing to delay treatment until they are very sick.

**Fig. 1:**
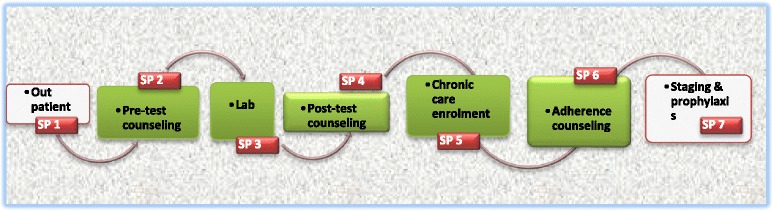
Service point (SP) for patients testing HIV positive on day of diagnosis within *gusta* clinics

Treatment delays resulting from clinical procedures were confirmed by our own observational data as illustrated in Fig. 1.

Before the move toward a global test-and-treat framework and the introduction of task shifting in Swaziland, individuals wanting to test for HIV would visit a voluntary counselling and testing (VCT) site, where they would receive pre- and post-test counselling, tested for HIV, and received test results, all from one health worker. As we have detailed elsewhere, [12], with task shifting, HIV testing entails five discrete steps, five queues, and often meeting with more than one health worker. Task shifting was meant to simplify processes and improve care; yet in practice it has contributed to the fragmentation of processes, which are now divided among multiple health workers.

The conversations I had with health workers who worked in HIV care delivery prior to the introduction of task shifting, indicate that the norm was for one health worker to administer the initiation of a patient into care at clinics in Swaziland. A patient usually only queued once per clinic visit, at most twice if including the collecting of medicines from the dispensary. According to nurses, the maximum time spent at a clinic should not exceed three hours. Yet, a patient testing for HIV must queue at up to seven service points in one day to complete the requisite steps associated with diagnosis. Our observations at the clinic recorded patients spending a minimum of three hours per clinic visit, with some patients spent up to six hours to complete the required steps. Thus task shifting not only multiplied the number of health workers a patient must see during a visit, but also extended waiting times. In addition, the current set-up also introduced greater possibilities for confidentiality breaches, as well as psychological burdens on patients who have to interact with multiple health workers on what is already a very stressful day for most.

Once the diagnosing processes are completed, those who test positive must embark on a second protracted journey to prepare for treatment initiation.

**Fig. 2:**
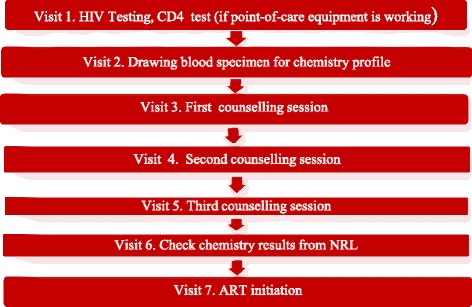
Clinic visits required to initiate ART at g*usta* clinics

Figure 2 illustrates the mandatory steps to be taken before ART can be initiated; no step can supplant another. Patient visits were profiled and validated through interviews, and found that under ideal circumstances a patient made no fewer than seven visits to the clinic to complete the ART preparation phase. This was regardless of whether a patient was very ill or asymptomatic. Neither a dangerously low CD4 count nor a patient’s desire to start treatment immediately or combine steps were taken into account. The only exceptions made in the guidelines were for children, pregnant women, or tuberculosis patients, who were generally started on ART immediately after diagnosis.

### Managing patients to manage data

The procedures and processes that accompanied treatment scale up and task shifting were introduced to streamline access to treatment and reorganise the health system to accommodate a rapidly growing number of patients. The steps illustrated in Fig. 1 were instituted to relieve nurses of the more menial tasks associated with preparing patients for treatment, including SP2, 4, 5, and 6. This was intended to allow nurses to focus on more technical tasks. It was also presumed that spreading the tasks among several staff members would minimise time spent by patients and make queues expedient at a particular service point and permit patients who came for a specific service to queue directly at the appropriate service point; for example, those coming for CD4 monitoring could go straight to the lab (SP3), those coming for an HIV test could go straight to the pre-ART room (SP2), and patients already on ART could go directly to the adherence monitoring room (SP6). When asked to explain the logic behind the system, one expert client said, it was an intentional strategy to manage the queues and prevent patients from being stuck in one line for a long time. Yet, there was also another reason for separating out the steps that patients followed: ease of data management and record keeping. Pre- and post-ART patients were required to report to separate service points and to use separate patient registers.

The seven mandatory clinic visits illustrated in Fig. 2 were instituted for several different reasons. First, according to the standard operating procedure for NARTIS, patients were supposed to receive their CD4 results on the same day of testing (Visit 1 in Fig. 2), which only became a possibility because the donor-funded project had provided a CD4 analysing machine known as PIMA and hired a phlebotomist to operate it in the *gusta* clinic. However, if the PIMA broke down, patients would have to return to the clinic at a later date to do the CD4 count. This sometimes meant more than one trip depending on how quickly the PIMA was fixed or a new PIMA installed. During the second clinic visit, patients had to give three tubes of blood to be sent to the national laboratory for blood chemistry analysis, a service that was not available at the primary health facilities. Each clinic was given specific days of the week for drawing blood and the collection of blood specimens for chemistry profiles to ensure the specimens reached the lab in good condition. If a client was diagnosed and became eligible for ART on a day that did not fall within the blood collection days, it meant the client had to return to the clinic on another day.

Beginning with the third clinic visit, patients begin a three-step counselling process meant to prepare them to make a lifelong commitment to ART adherence. The treatment guidelines stipulate that a client must attend three successive ART counselling sessions, with a minimum of seven days in-between sessions to assimilate and process information. If patients were able to return every week as planned, the pre-ART counselling phase would last 3 weeks at minimum. The three sessions were mandatory, regardless of the patient’s desire to start treatment sooner or a counsellor’s assessment of patient readiness.

An additional clinic visit was required to collect blood chemistry results. Patients were given a 2-week turnaround time before returning to the clinic to check their results from the national laboratory. However, some patients waited much longer than 2 weeks because samples sometimes spoilt during transportation from clinic to laboratory, which meant that a fresh specimen had to be taken and meant another 2 weeks in wait. Other delays were caused when; samples were lost or mixed up, when laboratory equipment at the national laboratory broke down, and when reagents were out of stock. During the research period, the country experienced a 4-month stock-out of reagents, and such was not uncommon. During that period it meant countrywide, no new patients could be started on treatment. Additionally, all patients who had given blood in the lead up to the stock-out had to return to the clinic to have fresh blood drawn (unpublished observation, December 2012). Obviously, such breakdowns and delays are bound to cause lack of trust in the system, as well as feelings of frustration and betrayal.

### Punitive care instead of patient empowerment

Treatment expansion has been characterised by a push to meet set targets that put a lot of pressure on health workers [13]. The *gusta* clinics were given targets to reach every month: number of people tested, enrolled into care, successfully tracked and linked to care, and adherence rates. In the *gusta* clinics, a report showing a decline in these statistics would be a nightmare for counsellors, which is expert clients. Because HIV counsellors stipend was paid by implementing partners^4^ to double up as clinic mentors on ART issues, they would be subjected to extensive questioning and expected to explain any decrease in uptake of services or anomalies in the data. Although implementation partners expected a lot in terms of monitoring and evaluation of programmes from the clinics where I conducted research, the burden was often heaviest for the counsellors who were held directly accountable. For example, some expert clients responsible for tracking defaulters reported that when allocated funds ran out, they would use their own funds to track patients who had failed to come back for a clinic visit. Although they were often concerned with client health outcomes, the reason they gave for going to these extreme measures was the pressure they endured to ensure their reports met the expectations of the implementation partners (unpublished observation, August 2013).

The pressure to attend to high patient loads meant that health care workers could spend even less time with patients than their already busy schedules allowed. From our observational data, we were able to calculate that on average nurses in both clinics saw up to 70 patients in a busy day during the first and last week of the month. The number was higher for expert clients because they also attended to patients who did not have to see the nurse, such as those receiving pre-test counselling (SP2) and those counselled in preparation for ART (SP6). In view of such realities, ethical issues and conduct, as well as quality of care were sometimes compromised in practice. Under immense time constraints and pressure to perform, expert clients sometimes passed their frustration onto patients. They were often authoritative and disciplining during counselling sessions.

During HTC training, counsellors were told that a good counselling session requires ‘a counsellor to have big eyes, big ears, and a small mouth’, meaning a good counsellor must be observant, pay attention to the patient in order to notice even bodily cues, and must listen more and talk less. However, the opposite was often observed in practice; counsellors dominated conversations and dictated to patients. Another principle stressed during the counselling training was that of confidentiality. Confidentiality depends on the counsellor having enough time in a private space to discuss the patient’s history and health status. In practice, patients were counselled in open and shared spaces; in one of the *gusta* sites, two counsellors shared one room and gave only standardised messages during counselling. Little if any attempt was made to create the context for an open and honest dialogue. Furthermore, it seemed that counselling messages were primarily intended to get patients to adhere to treatment at all costs rather than to support them as they attempted to deal with the complex social and cultural dynamics of an HIV infection or things that could potentially impede uptake of treatment (unpublished observation, June 2013). For example, one counselling session for a non-adherent patient seemed more like a court session, where the patient was treated as if he had broken a law and was punished through humiliating verbal reprimands. Utterances commonly used during counselling sessions included ‘we will send police to get you if you refuse to take treatment’ and ‘you will get a resistant strain of the virus if you don’t take medication properly, and die’. Such findings confirm similar trends identified by others researching counselling in the age of treatment scale-up [3, 14, 15] counsellors have become more concerned with regulating HIV patients’ sex lives and disciplining patients for breaking rules than empower.

### Shifting care constellations and challenged competencies

We observed that the pressure associated with treatment expansion-reaching targets, limited resources-coupled with burnout, gradually reduced the competence of nurses as they became less enthusiastic and less vigilant in their work. In an interview with an ART nurses, she said:

Back in the days when ART started, we did not take tea and lunch breaks as long as there was a patient waiting, we had to rotate, this is no longer the case. How much can one put in when you don’t get anything back? We work so hard and even on weekends as many things are added in our jobs because of HIV, but there is no incentive. They don’t even care that we need psychological support and debriefing as health workers. We used to have annual workshops in hotels, which gave us an opportunity to interact with our peers about our work in a more relaxed environment and a form of consolation. It was therapeutic for us. (Personal communication, November 2013)

Increased workload has been associated with reduced performance and less sensitivity towards patient needs of [16–18]. Maben [19] reports reduced emotional competence and interest when there are overwhelming organisational constraints. Not surprisingly, in our study nurses also reported that high patient numbers and organisational pressures related to record keeping, combined with frequent changes in ART protocols and procedures, resulted in despondency and complacency. Health care workers also reported feeling disempowered in the context of increasing and sometimes conflicting demands from donors and the State. One ART nurse stated:

They tell us how to do things and sometimes we are confused because we get different instructions. ICAP says this, World Vision says that, and the Ministry of Health the other thing. So we wait until they discuss and get direction from them because with HIV our discretion is limited. There is a lot we do not know, there are many changes, and we have to rely on guidance. (personal communication, February 2013)

Much of the work performed by nurses within the ART programme was learnt on the job rather than in formal training. The avalanche of protocols and dependence on mentors for technical support in provision of care limited nurses’ ability to apply discretion in providing care and resulted in limited competence. In some cases, however, the nurses simply lacked sufficient training. The first author witnessed patients given the wrong treatment or turned away because the nurse was unsure how to intervene. Also in one case, a patient presented with a condition that was a side effect of one of the ARV drugs she was taking. Although the expert client knew about the problem, the nurse did not.

Generally expert clients had greater competence than nurses when it came to taking HIV medicines. They knew the different types of pills by name and sight and quickly noticed if a client had received the wrong medication. Due to the increase in such errors, expert clients in the clinic made it their prerogative to check the patients’ medicines before they left the clinic. Such practices shaped the patients’ experience dramatically. Expert clients were increasingly positioned to serve patients better than nurses, taking up some of the technical tasks that were left for clinical acumen. The danger of patients being given the wrong pills due to the under-training and over-working of nurses cannot be over-emphasised. Beyond that, nurses experienced a decrease in power at the expense of expert clients, which sometimes translated to tensions in the workplace, see also [20, 21].

## Discussion

As conceptualised, task shifting was intended to resolve human resource challenges needed to expand treatment, to ensure the sustainability of programmes, and to improve quality of care of patients through the simplification of processes and the provision of patient-friendly services [8]. Our findings suggest that while task shifting has facilitated the scale-up of treatment, it may actually decrease the quality of care and has certainly made clinical processes more complex. Task shifting multiplied service points as tasks were delegated to lower cadres. Consequently, patients spent a lot of time at the clinic on the day of diagnosis. Long queues were the norm in the clinics where this research was conducted. Health workers had to be practical and prioritise seeing everyone in the queues even when it meant compromising quality and fundamental principles. The pressure on health workers to deliver within these conditions caused health workers to pass the pressure onto patients. Therefore, care became disciplinary rather than empowering, particularly for patients who did not adhere to protocols. Certainly, globally derived policies influence modalities of care delivery at the local level as well as shape patients’ experiences. We coin the term *policypeutic* to capture how policies have intrinsic meaning and are relationally experienced and embodied by the intended users.

Furthermore, the derived policies do not always come with all the support required for the strategies to work efficiently. Efforts to rapidly expand treatment were made by bringing comprehensive services to the grassroots level (i.e., boost HR capacity and bring equipment), however there were many logistical challenges encountered by the clinics: spoilage of blood samples, breakdown of equipment, delays in tests results. Due priority was not given in resolving these interruptions which were key to the treatment scale-up plan. This became visible during the four-month stockout of reagents that occurred during the course of the study, yet more patients were enrolled whilst many were lost as their results did not return from the national referral laboratory.

In ideal circumstances, a compliant patient in Swaziland waits an average of 2 months before treatment begins due to mandatory procedures. Long waits and bureaucracy can be a disincentive for eligible patients [22]. The new systems and guidelines were applied without discretion: timelines and protocols outweighed patients’ concerns and limited the agency of nurses in particular. Although never the intention of implementing agencies, strict protocols and programmatic audit obligations pressured health workers to prioritise those matters over saving lives or client-friendly services. Moreover, treatment expansion was implemented amidst a cocktail of institutional constraints: staff shortages, limited infrastructure, and constantly shifting guidelines. It was simply impractical for health workers to provide the gold standard of care under such circumstances. The fragmentation of care that resulted from task shifting and other policy guidelines only heightened this frustration.

However, measured against project-determined ‘audit rituals’ [23, 24], the MaxART project was a success. As Crane [25] has argued, success of interventions should not only be based on evidence that is sanctioned by those who decide what to include and exclude in knowledge production. MaxART reached and surpassed its own HIV testing target within a year of implementation, whilst the retention levels and treatment rates also moved closer to the target within a year of implementation [26]. The success was based on arithmetic models: tests done, equipment and pamphlets distributed, trainings, meetings conducted, and attendance of the meetings. But what is concealed in the reports are patient and health care provider experiences of the processes and quality of care dynamics that came with task shifting.

Task shifting has unquestionably alleviated the acute human resource situation and facilitated the massive expansion of HIV treatment programmes in Swaziland and across Africa. But it was never going to be a permanent fix for the continent’s human resource issues. Africa produces a meagre 5,100 medical doctors per annum [8]. Addressing these issues will require serious long-term investment [27] even to meet the suggested minimum ratios for HIV treatment: two physicians, seven nurses, three pharmacy staff, and a large pool of lay workers for every 1,000 HIV patients [28]. Instead of providing sustainable solutions, funding agencies resort to temporary, low-cost, and unsustainable options such as minimally funding volunteers and providing technical assistance to government. Furthermore, the Ministry of Health’s 2010 National AIDS Spending Assessment report [29] shows that although 57% of national AIDS expenditure is funded by donor agencies, the majority of the donor funds goes to overhead costs, project management, and administration whilst the state must cover remaining capital expenses, procurement of ARVs, maintenance of clinics, payment of health workers and provision of infrastructure. In the long run, such globally derived initiatives exert a lot of pressure and weaken already fragile public health system [30].

## Conclusion

Task shifting becomes a stopgap solution that leads neither to sustainability or the strengthening of health systems, nor to the accelerated training of health workers required to meet the national demands of the ART programme. Therefore strengthening of the health system should be a priority and task shifting is one such a strategy that needs to be implemented, to compliment treatment expansion efforts. That said, promotion of new strategies need to foster positive therapeutic experiences and better outcomes for patients than just purely focusing on arithmetic targets. The authors recommend that evaluation of HIV programmes and interventions should incorporate metrics that can account for the quality of services and also take into account the views and experiences of on-the-ground, healthcare workers and patients. The case of Swaziland serves as an important example for other countries attempting to scale-up HIV treatment rapidly. Although other countries in Africa and elsewhere have been expanding access to treatment over the last decade, the Swazi ‘experiment,’ offers insights into the dangers of emphasizing reaching short-term targets that might affect issues of quality service delivery and long-term sustainability. Given the immense funding and technical expertise funnelled into Swaziland in a very short period of time, the recent national effort to scale-up treatment is somewhat unique, especially given that HIV testing and treatment rates were so low in Swaziland prior to the recent push. Given the focus of this article, we refrain from examining the Swazi “success” in comparison to other countries. Instead, we have tried to highlight some of the ‘failures’ concealed by the Swazi success story.

Although Swaziland has managed to get many more people in need to test for HIV and to enrol in treatment programmes in a very short period of time, it remains to be seen if the health system can sustain the level of success it has demonstrated, if people can be kept on treatment over the course of their lives, or if overburdened healthcare workers can be supported in their careers, both materially and psychologically. HIV treatment is for life, the life of those infected, as well as the life of those providing care. Swaziland may be winning in the sprint competition, but the marathon lies ahead.

The Swazi experiment also offers insights into the dangers of the bureaucratization of HIV care delivery. The Kafkaesque proliferation of service points in Swaziland has actually increased the length of time people have to wait for services on any given day and also the time between finding out one’s HIV status and being enrolled in a treatment programme. This has created frustrations, economic expense, and psychological distress for patients. Although we support the aim of collecting evaluation data to improve services, we question the motivations of donors who insist on making treatment programmes more complex to allow them to meet the demands of internal organizational monitoring and evaluation frameworks. Finally, given the very important role that expert clients play in filling the multiple gaps in HIV care provision, we argue that national health ministries should move to regularize this cadre of health workers to ensure continued employment for them and the sustainability of treatment programmes as a whole.

## Abbreviations

ARVs: Antiretrovirals
CD4: Cluster of Differentiation 4
CHAI: Clinton Health Access Initiative
HPTN: HIV Prevention Trials Network
HTC: HIV Testing and Counselling
ICAP: International Centre for AIDS Care and Treatment
MaxART: Maximizing ART for zero new infections
NARTIS: Nurse-led ART Initiations
NERCHA: National Emergency Response Council on HIV/AIDS
SP: Service Point

## Footnotes

1 MaxART is a programme funded primarily by the Dutch National Postcode Lottery. Collaborating partners included the Clinton Health Access Initiative, Stop AIDS Now, the Swaziland Ministry of Health, SafAIDS, the Swaziland Network of People Living with HIV/AIDS (SWANEPHA), the South African Centre for Mathematical Modeling (SACEEMA), the Global Network of People Living with HIV/AIDS (GNP+), and the University of Amsterdam. The University of Amsterdam was the lead social science research partner; the authors of this paper were members of the social science team from that university. Swaziland was the first country to pilot an intervention based on recommendations from the HPTN 052 trial.

2 Expert clients and lay health workers are different in the context of the study; lay health workers are PLHIV and their role is limited to providing counselling. Expert clients are also PLHIV on ART, but they are hired as full time health workers in health settings, they double as HIV counsellors and assume tasks ‘shifted’ from those previously taken up by nurses. They include pre- and post-test counselling, the enrolment of patients into care, adherence counselling for patients on ART, and patient tracking among other things.

3 Previous research on task shifting has focused on its efficacy and usefulness [3–5, 31] as well as challenges for incorporating a new non-medically trained cadre within biomedical spaces [6, 14, 20, 21, 32–35].

4 Mainly ICAP, Global Fund, and World Vision.
